# Estimation of populations at risk for severe influenza and vaccination coverage in Mexico, 2010–2021

**DOI:** 10.3389/fepid.2026.1826780

**Published:** 2026-07-21

**Authors:** Arturo Galindo-Fraga, Gerardo Luna-Casas, Ricardo Gasca-Pineda, Gilberto Sánchez-González

**Affiliations:** 1 Epidemiology Department, National Institute of Medical Sciences and Nutrition Salvador Zubirán, Mexico City, Mexico; 2 Estimatio, Health Economics and Outcome Research Services, Mexico City, Mexico; 3 Sanofi Vaccines, Mexico City, Mexico; 4 Center for Research on Infectious Diseases, National Institute of Public Health, Cuernavaca, Morelos, Mexico

**Keywords:** at-risk populations, epidemiology, influenza, Mexico, vaccination

## Abstract

**Background:**

Influenza remains an important cause of morbidity among populations with underlying medical conditions associated with increased risk of severe disease. This study aimed to estimate the size of populations eligible for influenza vaccination according to the Mexican Universal Vaccination Program and to evaluate vaccination coverage gaps among populations at risk in Mexico between 2010 and 2021.

**Methods:**

A retrospective analytical study was conducted using epidemiological and administrative healthcare databases from Mexican public healthcare institutions. Population estimates were constructed using prevalence-based epidemiological projections, healthcare system records, and attended patient data from populations at risk. Descriptive analyses and time-series modeling were performed to evaluate vaccination coverage patterns and healthcare demand over time.

**Results:**

Across all analyzed populations at risk, the number of individuals receiving healthcare services was consistently lower than the number of notified cases and substantially lower than prevalence-based epidemiological estimates. Vaccination coverage varied considerably across populations at risk and remained incomplete throughout the study period. In 2021, although approximately 12.5 million influenza vaccine doses were administered among populations at risk, a substantial proportion of potentially eligible individuals remained unvaccinated. Forecasting analyses suggested a progressive increase in healthcare demand among populations at risk over time.

**Conclusions:**

Important gaps persist between the estimated population at risk, diagnosed individuals, healthcare utilization, and influenza vaccination coverage in Mexico. The analytical framework proposed in this study integrates epidemiological prevalence estimates, healthcare utilization patterns, and vaccination data to identify unmet vaccination needs and may support improved public health planning, prioritization strategies, and strengthening of influenza vaccination programs in Mexico and similar settings.

## 1 Introduction

Influenza circulates worldwide with marked seasonal patterns and, depending on disease severity, may lead to hospitalization and death (1). The 2022–2023 influenza season resulted in a high number of cases in several countries, including the United States [(2), where annual influenza vaccination is universally recommended (3)]. Influenza vaccination has been shown to reduce severe disease, hospitalizations, and mortality, and may also contribute to reducing transmission rates (4–7). In this context, preliminary estimates from the Centers for Disease Control and Prevention (CDC) indicated that during the 2022–2023 season in the United States, an estimated 26–54 million influenza cases occurred, resulting in 12–26 million medical visits, 290,000–640,000 hospitalizations, and 18,000–57,000 deaths (data available through April 29, 2023) (6).

In Mexico, according to Ministry of Health reports, 9,877 influenza cases were registered during the 2022–2023 season, representing the highest number reported since the 2009 influenza pandemic (7). Individuals aged 20–29 years accounted for 22% of all reported cases, while those aged 30–39 years represented 17%. These figures correspond to the national sentinel surveillance system for viral respiratory diseases (8).

The Mexican Universal Vaccination Program (Programa de Vacunación Universal, PVU) recommends annual influenza vaccination for several priority populations, including children aged 6–59 months, pregnant women, adults aged ≥60 years, healthcare workers, and individuals aged 5–59 years living with selected at-risk conditions (9). The routine immunization schedule includes two doses administered during the first year of life beginning at 6 months of age, separated by at least one month.

According to the national influenza vaccination guidelines for the 2022–2023 season, the population at risk includes individuals with congenital or chronic cardiovascular and pulmonary diseases, conditions associated with prolonged salicylate use in children and adolescents, diabetes mellitus, morbid obesity (BMI >40 kg/m^2^), chronic lung disease including asthma and chronic obstructive pulmonary disease (COPD), cardiovascular disease excluding essential hypertension, chronic kidney disease, immunosuppression due to disease or treatment, cancer, and people living with HIV/AIDS (9). These populations are considered at increased risk of influenza-related complications, hospitalization, and mortality, and therefore constitute priority groups for seasonal influenza vaccination in Mexico.

Each year, the Ministry of Health estimates the number of influenza vaccine doses required for the vaccination season. For populations at risk, the number of doses has historically been estimated as approximately 5% of the population aged 5–59 years (10). Therefore, a scenario may exist in which the number of individuals belonging to populations at risk exceeds the number of available vaccine doses.

The objective of this study was to estimate the size of populations eligible for influenza vaccination among populations at risk in Mexico, assess vaccination coverage in these groups, and explore whether the current operational estimates used for vaccine allocation adequately reflect the potential population at risk during the period 2010–2021.

## 2 Materials and methods

### 2.1 Study design and analytical framework

A retrospective, descriptive, and analytical database study was conducted using surveillance and administrative healthcare databases obtained from public healthcare institutions in Mexico. The primary analytical period covered 2015–2021; however, time-series analyses were additionally extended to the period 2010–2021 to evaluate temporal trends and forecast healthcare demand.

For analytical purposes, the study population was categorized according to the operational definitions established in the Mexican Universal Vaccination Program (Programa de Vacunación Universal, PVU), including the target population (TP) and the population at risk (PAR). Additional operational epidemiological categories used throughout the analysis included estimated cases (EC), notified cases (NC), attended patients (AP), persons vaccinated (PV), and personnel at risk (PR), the latter corresponding exclusively to healthcare workers included in vaccination coverage calculations.

**Figure 1:**
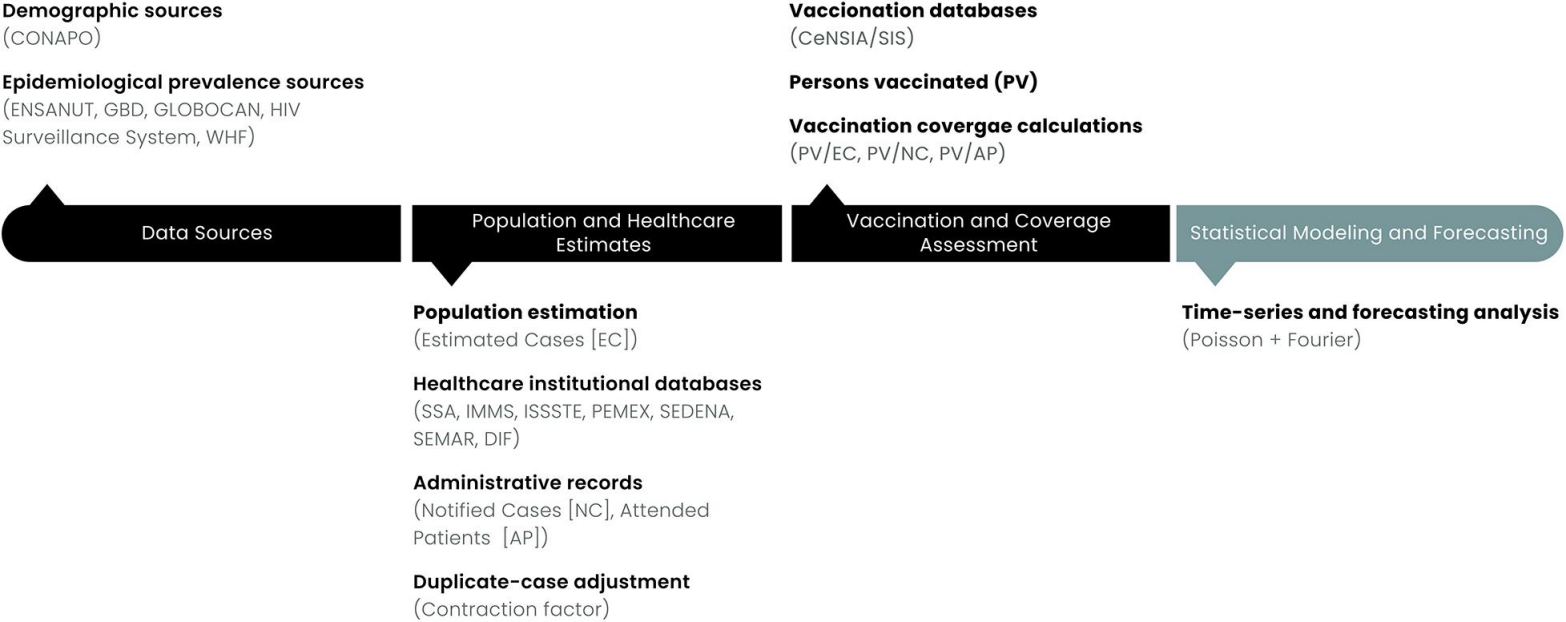
Analytical framework and data-processing workflow used for population estimation, vaccination coverage calculations, and forecasting analyses. Flow diagram illustrating the integration of demographic projections, epidemiological prevalence sources, healthcare institutional databases, vaccination registries, and analytical procedures used to estimate populations at risk, vaccination coverage indicators, and forecasting analyses in Mexico during 2010–2021. CONAPO, National Population Council; ENSANUT, National Health and Nutrition Survey; GBD, Global Burden of Disease; WHF, World Heart Federation; EC, estimated cases; NC, notified cases; AP, attended patients; PV, persons vaccinated; CeNSIA, National Center for Child and Adolescent Health; SIS, Health Information System; SSA, Ministry of Health; IMSS, Mexican Social Security Institute; ISSSTE, Institute for Social Security and Services for State Workers; PEMEX, Petróleos Mexicanos healthcare services; SEDENA, Ministry of National Defense; SEMAR, Ministry of the Navy; DIF, National System for Integral Family Development.

The overall analytical framework, data sources, and processing workflow used for population estimation, vaccination coverage calculations, and forecasting analyses are illustrated in Figure 1.

### 2.2 Data sources

#### 2.2.1 Demographic population estimates

Population projections were obtained from databases maintained by the Mexican National Population Council (Consejo Nacional de Población, CONAPO), which provide annual demographic estimates of the size and structure of the Mexican population by age, sex, and state (11). Data corresponding to the period January 2010 to December 2021 were extracted and stratified into the following age groups: <5 years, 5–9 years, 10–19 years, 20–49 years, 50–59 years, and ≥60 years.

#### 2.2.2 Influenza vaccination data

Information on influenza vaccine doses administered was obtained through requests submitted to the Transparency Unit (TU) of the National Center for Child and Adolescent Health (CeNSIA) through the National System of Transparency, Access to Public Information and Protection of Personal Data (INAI) (12).

Reports from the Health Information System (Sistema de Información en Salud, SIS) corresponding to the period 2015–2021 were obtained. These reports included information on influenza vaccine doses administered to the target population (TP), including first doses administered to children aged 6–59 months, second doses administered to children aged 7–59 months, and annual revaccination doses administered to eligible adults aged 18–59 years and adults aged ≥60 years. Additional records included vaccine doses administered to pregnant women, healthcare workers, and populations with selected at-risk conditions.

Vaccination data were obtained by age group, federative entity, and healthcare institution, including the Ministry of Health (SSA), the Mexican Social Security Institute (IMSS), IMSS-Bienestar, the Institute for Social Security and Services for State Workers (ISSSTE), Petróleos Mexicanos healthcare services (PEMEX), the Ministry of National Defense (SEDENA), the Ministry of the Navy (SEMAR), the National System for Integral Family Development (DIF), and other state or municipal healthcare institutions.

#### 2.2.3 Population at risk and comorbidity data

Information on healthcare demand among populations at risk (PAR) was obtained through requests submitted to the Transparency Units of public healthcare institutions.

According to the national influenza vaccination guidelines, the analyzed populations at risk included pregnant women, healthcare workers, and individuals living with selected comorbidities associated with severe influenza outcomes. Healthcare workers included physicians, nurses, paramedics, healthcare students with direct patient contact, administrative personnel working in clinical areas, pharmacy personnel, and other frontline healthcare staff.

The analyzed comorbidities within the population at risk included diabetes mellitus, morbid obesity (BMI ≥40 kg/m^2^), cardiovascular disease excluding essential hypertension, chronic lung disease including asthma and chronic obstructive pulmonary disease (COPD), congenital or chronic cardiovascular and pulmonary diseases, chronic kidney disease, cancer, immunosuppression due to disease or treatment, autoimmune and hematological conditions associated with immunosuppression, conditions associated with prolonged salicylate use in children and adolescents, and people living with HIV/AIDS.

Requested variables included ICD-10 codes, age groups, federative entity, healthcare institution, and annual records corresponding to the period January 2010 to December 2021. Information was obtained from SSA, IMSS, IMSS-Bienestar, ISSSTE, PEMEX, SEDENA, SEMAR, DIF, and other state or municipal healthcare institutions.

### 2.3 Population estimation framework

Vaccination Program (PVU), literature-based prevalence estimates were applied to broader administrative categories to estimate populations with uncontrolled diabetes mellitus and morbid obesity. Vaccinated individuals identified within these broader categories were assumed to correspond to the vaccination-eligible subpopulations defined by PVU criteria.

Prevalence estimates used to construct estimated cases (EC) were obtained from national and international epidemiological sources according to each analyzed condition. These included the National Health and Nutrition Survey (ENSANUT), a nationally representative population-based survey conducted by the National Institute of Public Health in Mexico using a probabilistic multistage sampling design, as well as the Global Burden of Disease Study (GBD), the World Heart Federation, GLOBOCAN, and national epidemiological reports.

Attended patients (AP) were defined as individuals with at least one healthcare encounter during a given year, without duplication, according to official outpatient healthcare records. This category represented the fraction of notified cases actively utilizing healthcare services within the public healthcare system and was considered the lower operational boundary of the population potentially reachable through vaccination programs.

The complete list of epidemiological sources and extracted prevalence estimates used for each analyzed condition is presented in Table 1.

**Table 1:** Sources and data extracted for prevalence estimates among populations at risk.

Population at risk	Epidemiological source	Key prevalence estimates used for population calculations
People living with HIV/AIDS	HIV Epidemiological Surveillance System 2010–2021 (28)	A total of 331,437 cumulative HIV cases had been identified through 2021. Among all reported cases, 62.52% corresponded to individuals living with HIV, and 95.6% of cases were observed among individuals aged 5–59 years.
Diabetes mellitus	ENSANUT prevalence estimates (29, 30); uncontrolled DM prevalence (31)	Diagnosed DM prevalence among individuals aged ≥20 years ranged from 9.2% to 10.3%, while undiagnosed DM prevalence ranged from 3.8% to 4.9%, resulting in an estimated total prevalence of 13.7%. Among individuals aged 10–19 years, diagnosed DM prevalence was 0.60% in males and 0.80% in females. The estimated proportion of uncontrolled DM cases was 68.3%.
Morbid obesity	ENSANUT obesity prevalence estimates (30); morbid obesity prevalence study (32)	Obesity prevalence among children aged 5–11 years ranged from 17.4% to 23.8% in males and from 11.8% to 15.0% in females. Among adolescents aged 12–19 years, prevalence ranged from 14.5% to 21.5% in males and from 12.1% to 15.0% in females. In adults aged ≥20 years, obesity prevalence ranged from 26.8% to 31.8% in males and from 37.1% to 41.1% in females. Estimated morbid obesity prevalence ranged from 2.9% to 3.6%.
Cardiovascular disease	World Heart Federation/World Heart Observatory/Global Impact CVD Mexico 2010–2021 (33)	Cardiovascular disease prevalence among individuals aged 5–14 years was estimated at 0.5% for both males and females. Among individuals aged 15–49 years, prevalence ranged from 1.5% to 1.7% in males and from 2.4% to 2.5% in females. In the 50–54 years age group, prevalence ranged from 5.8% to 6.2% in males and from 7.0% to 7.1% in females, while in individuals aged 55–59 years prevalence ranged from 9.0% to 9.4% in males and from 9.8% to 10.0% in females.
Chronic lung disease including asthma and COPD	Global Burden of Disease Study 2017 (29)	The estimated prevalence of chronic lung diseases, including asthma and COPD, was 6.14%.
Cancer	GLOBOCAN Mexico Factsheet 2020 (34)	The estimated 5-year cancer prevalence was 0.41%.
Congenital or chronic cardiovascular and pulmonary disease, including conditions associated with prolonged salicylate use	Global Burden of Disease Study 2017 (29)	The estimated prevalence of congenital or chronic cardiovascular and pulmonary diseases and conditions associated with prolonged salicylate use was 2.8%.
Chronic kidney disease	Epidemiological Profile of Chronic Kidney Disease in Mexico 2017 (35)	Chronic kidney disease prevalence ranged from 5,331.01 to 6,293.73 cases per 100,000 inhabitants.
Disease- or treatment-acquired immunosuppression	Global Burden of Disease Study 2017 (29)	The estimated prevalence of disease- or treatment-acquired immunosuppression was 4.4%, including dermatological, gastrointestinal, and musculoskeletal diseases associated with immunosuppressive conditions or treatments.

HIV/AIDS, human immunodeficiency virus/acquired immunodeficiency syndrome; DM, diabetes mellitus; COPD, chronic obstructive pulmonary disease; ENSANUT, national health and nutrition survey.

#### 2.3.2 Healthcare worker population estimates

Information on healthcare workers was obtained from the interactive databases of the General Directorate of Health Information (DGIS) (13). Annual estimates of healthcare personnel corresponding to the period 2010–2021 were extracted by healthcare institution and federative entity.

The analyzed population included physicians, nurses, paramedics, healthcare trainees in direct contact with patients, pharmacy personnel, administrative personnel working in clinical areas, and other frontline healthcare workers considered eligible for seasonal influenza vaccination under national vaccination guidelines.

#### 2.3.3 Vaccination coverage calculations

Vaccination coverage estimates were calculated by integrating the number of persons vaccinated (PV) with the operational population categories defined in this study. Coverage estimates were evaluated using three complementary epidemiological approaches: the ratio of vaccinated persons to estimated cases (PV/EC), the ratio of vaccinated persons to notified cases (PV/NC), and the ratio of vaccinated persons to attended patients (PV/AP).

These complementary indicators were used to evaluate potential differences between the theoretically eligible population derived from prevalence estimates, the population registered within healthcare system databases, and the population actively receiving healthcare services.

All population and vaccination coverage estimates were subsequently stratified by age group, healthcare institution, federative entity, and study year for all analyzed populations at risk.

### 2.4 Operational definitions and estimation approach

For analytical purposes, the study population was categorized into the target population (TP) and the population at risk (PAR), according to the definitions established by the Mexican Universal Vaccination Program (PVU).

The TP included routinely vaccinated groups, such as children aged 6–59 months, pregnant women, adults aged ≥60 years, and healthcare workers. The PAR included individuals aged 5–59 years living with selected conditions associated with severe influenza outcomes.

Four operational epidemiological categories were used throughout the study. Estimated cases (EC) corresponded to prevalence-based population estimates derived from demographic projections and epidemiological prevalence data. Notified cases (NC) referred to individuals with a documented diagnosis registered within healthcare system databases. Attended patients (AP) were defined as individuals with at least one healthcare encounter during a given year, without duplication. Persons vaccinated (PV) corresponded to individuals registered as having received at least one influenza vaccine dose during the analyzed vaccination season.

### 2.5 Adjustment for duplicate cases

To reduce potential double counting caused by overlapping comorbidities, administrative records presenting overlapping diagnoses across at-risk conditions included in the analysis were adjusted using a contraction-factor approach. This filtering method was evaluated using institutional databases containing sufficiently detailed information to identify overlapping records. The methodology is illustrated in Table 2 using morbid obesity cases identified after filtering overlapping comorbidities during 2021 as an example.

**Table 2:** Example of the duplicate-adjustment method applied to overlapping comorbidities (morbid obesity, 2021).

Patients with obesity-related ICD-10 records (2021)	Cases
Total attended patients with obesity-related ICD-10 records (A)	27,119,580
Non-duplicated attended patients with obesity-related ICD-10 records (B)	26,091,576
Non-duplicated attended patients with morbid obesity (C)	2,899,064
Contraction factor for morbid obesity (C/A)	0.106

The filtering strategy was feasible in 34 of the 76 healthcare institutions that provided the requested information, representing approximately 78% of the total attended patients included in the final database. The observed proportion of duplicate records was subsequently applied to the remaining institutions to minimize potential overestimation caused by repeated registrations.

An adjustment factor was calculated using institutional datasets containing patient-level identification variables that allowed the detection of overlapping comorbidities. The contraction factor for each analyzed condition was estimated as:

$$CF_{\mathrm{condition}}=\frac{N_{\mathrm{elegible},\mathrm{non}\text{-}\mathrm{duplicated}}}{N_{\mathrm{administrative}\;\mathrm{records}}}$$

where $CF_{\mathrm{condition}}$ represents the contraction factor for each analyzed condition, $N_{\mathrm{elegible},\mathrm{non}\text{-}\mathrm{duplicated}}$ corresponds to the number of non-duplicated individuals meeting PVU eligibility criteria after filtering overlapping comorbidities, and $N_{\mathrm{administrative}\;\mathrm{records}}$ represents the total number of administrative healthcare records initially identified before duplicate adjustment.

The adjusted number of attended patients was subsequently estimated as:

The adjusted number of attended patients $\left(AP_{\mathrm{adjusted}}\right)$ was subsequently estimated as:

$$AP_{\mathrm{adjusted}}=AP_{\mathrm{observed}}\times CF_{\mathrm{condition}}$$

where $AP_{\mathrm{observed}}$ represents the total number of attended patients initially identified through administrative healthcare records before duplicate adjustment, and $AP_{\mathrm{adjusted}}$ corresponds to the estimated number of attended patients after filtering overlapping comorbidities and applying PVU eligibility criteria.

### 2.6 Variables requested from healthcare institutions

Information requests submitted to healthcare institutions included ICD-10 codes corresponding to each analyzed condition, age group, federative entity, and annual records for the study period January 2010 to December 2021.

The analyzed ICD-10 groups included people living with HIV/AIDS (B20–B24), diabetes mellitus (E10–E14), obesity (E66), cardiovascular disease (I00–I99 excluding I10X), chronic respiratory diseases including asthma and COPD (J40–J47), cancer (C00–D48), congenital or chronic cardiovascular and pulmonary diseases (Q20–Q28 and Q30–Q34), chronic kidney disease (N17–N19), disease- or treatment-acquired immunosuppression (G60–G64, G70–G73, K50–K52, and M00–M99), and pregnancy (Z34.9).

The complete list of variables and ICD-10 definitions included in the requests submitted to Transparency Units is presented in Table 3.

**Table 3:** Variables included in requests for information to transparency units.

Variables	Description
Population at risk using ICD-10 Codes	▪ People living with HIV/AIDS (ICD-10: B20–B24). ▪ Diabetes mellitus (ICD-10: E10–E14). ▪ Obesity (ICD-10: E66). ▪ Cardiovascular disease [ICD-10: I00–I99, excluding essential hypertension (I10X)]. ▪ Chronic respiratory diseases, including asthma and COPD (ICD-10: J40–J47). ▪ Malignant neoplasms and other cancers (ICD-10: C00–D48). ▪ Congenital heart and lung diseases, and conditions associated with long-term salicylate therapy (ICD-10: Q20–Q28 and Q30–Q34) ▪ Chronic kidney disease (ICD-10: N17–N19). ▪ Acquired or treatment-related immunosuppression (ICD-10: G60–G64, G70–G73, K50–K52, and M00–M99). ▪ Pregnancy (ICD-10: Z34.9)
Age group	▪ 5–9 years ▪ 10–19 years ▪ 20–49 years ▪ 50–59 years
Federative entities	All 32 federative entities of Mexico.
Study period	January 2010 to December 2021

HIV/AIDS, human immunodeficiency virus/acquired immunodeficiency syndrome; COPD, chronic obstructive pulmonary disease; ICD-10, international classification of diseases, tenth revision.

### 2.7 Statistical analysis

Descriptive statistics were calculated for all analyzed populations at risk, vaccination categories, and age groups. Time-series analyses of comorbidity cases during the period 2010–2021 were performed using Poisson regression models adapted to the observed data. Regression analyses evaluated secular trends (month and year) and seasonal variation using Fourier terms.

The general Poisson model used in the analysis was:

$$\ln[E(y_{i})]=\beta_{i0}+\sum_{\;j=1}^{K}\beta_{ij}x_{j}$$

where $y_{i}$ represented the number of observed cases, $\beta_{ij}$ represented the regression coefficients, and $x_{j}$ represented the regression variables included in the model.

The seasonal component was modeled using a sine function:

$$\mathrm{seasonality}=\sin^{2}\left(\frac{2\pi t}{12}\right)$$

where *t* represented time measured in months.

To estimate population and vaccination coverage outcomes across the age groups of interest, population estimates by age group obtained from the National Population Council (CONAPO) database were used as denominators.

Forecasting models were subsequently applied to estimate future healthcare demand among populations at risk. All statistical analyses were performed using Stata version 15 (StataCorp, College Station, TX, USA).

## 3 Results

### 3.1 Prevalence of populations at risk and influenza vaccination coverage in Mexico

Marked differences were observed in the epidemiological burden, attended patients, and influenza vaccination coverage among the populations at risk included in the Mexican Universal Vaccination Program during the study period (see Table 4).

**Table 4:** Prevalence, attended population, and influenza vaccination coverage among populations at risk in Mexico, 2015–2021.

Population at risk	2015	2016	2017	2018	2019	2020	2021
HIV/AIDS							
NC	143,032	151,314	160,235	170,426	180,707	186,583	194,797
Prevalence (%)	0.15	0.15	0.16	0.17	0.18	0.18	0.19
AP	103,826	108,080	127,695	120,639	154,341	152,012	159,444
PV	56,445	49,347	72,159	64,525	55,989	70,660	89,382
PV/AP (%)	54.4	45.7	56.5	53.5	36.3	46.5	56.1
PV/NC (%)	39.5	32.6	45.0	37.9	31.0	37.9	45.9
Diabetes mellitus							
EC	6,601,885	6,853,030	7,224,822	7,628,320	7,596,667	7,534,422	7,514,614
EC prevalence (%)	6.70	6.90	7.20	7.60	7.50	7.30	7.30
NC	4,599,428	4,787,044	4,967,864	5,169,191	5,153,137	5,109,867	5,109,938
NC prevalence (%)	4.70	4.80	5.00	5.10	5.10	5.00	4.90
AP	3,120,808	3,199,844	3,294,169	3,735,918	3,993,072	4,417,966	4,523,526
PV	1,712,394	2,063,285	2,108,741	2,445,869	2,639,817	3,010,320	1,802,372
PV/AP (%)	54.9	64.5	64.0	65.5	66.1	68.1	39.8
PV/NC (%)	37.2	43.1	42.4	47.3	51.2	58.9	35.3
PV/EC (%)	25.9	30.1	29.2	32.1	34.7	40.0	24.0
Uncontrolled diabetes mellitus							
EC	4,502,486	4,673,766	4,927,329	5,202,514	5,180,927	5,138,476	5,124,967
EC prevalence (%)	4.60	4.70	4.90	5.20	5.10	5.00	5.00
NC	3,136,810	3,264,764	3,388,083	3,525,388	3,514,439	3,484,929	3,484,978
NC prevalence (%)	3.20	3.30	3.40	3.50	3.50	3.40	3.40
AP	2,128,391	2,182,294	2,246,623	2,547,896	2,723,275	3,013,053	3,085,045
PV	1,712,394	2,063,285	2,108,741	2,445,869	2,639,817	3,010,320	1,802,372
PV/AP (%)	80.5	94.5	93.9	96.0	96.9	99.9	58.4
PV/NC (%)	54.6	63.2	62.2	69.4	75.1	86.4	51.7
PV/EC (%)	38.0	44.1	42.8	47.0	51.0	58.6	35.2
Morbid obesity (BMI ≥40 kg/m^2^)							
EC	2,634,807	2,759,025	2,931,906	3,327,771	3,378,736	3,581,330	3,727,302
Prevalence (%)	2.70	2.80	2.90	3.30	3.30	3.50	3.60
AP	2,100,185	2,004,947	1,721,214	2,187,692	2,238,117	3,442,586	2,899,064
PV	1,625,690	1,545,114	1,331,632	1,704,587	1,735,413	2,664,670	2,696,306
PV/AP (%)	77.4	77.1	77.4	77.9	77.5	77.4	93.0
PV/EC (%)	61.7	56.0	45.4	51.2	51.4	74.4	72.3
Cardiovascular disease							
EC	2,549,187	2,603,564	2,713,874	2,759,136	2,798,874	2,843,009	2,885,948
Prevalence (%)	2.60	2.60	2.70	2.70	2.80	2.80	2.80
AP	791,523	744,159	765,398	940,730	942,389	1,482,236	1,221,288
PV	540,729	436,110	472,679	583,447	579,053	945,039	915,422
PV/AP (%)	68.3	58.6	61.8	62.0	61.4	63.8	75.0
PV/EC (%)	21.2	16.8	17.4	21.1	20.7	33.2	31.7
Chronic lung disease							
EC	5,916,553	6,033,299	6,144,621	6,250,785	6,352,032	6,448,172	6,539,008
Prevalence (%)	6.00	6.10	6.10	6.20	6.20	6.30	6.30
AP	1,917,948	1,475,841	1,217,795	1,408,113	1,460,687	2,104,425	1,468,618
PV	735,213	655,218	526,285	615,503	634,394	899,003	1,069,379
PV/AP (%)	38.3	44.4	43.2	43.7	43.4	42.7	72.8
PV/EC (%)	12.4	10.9	8.6	9.8	10.0	13.9	16.4
Cancer							
EC	407,735	411,738	415,519	419,093	422,474	425,659	428,647
Prevalence (%)	0.40	0.40	0.40	0.40	0.40	0.40	0.40
AP	293,397	171,478	212,917	251,374	261,211	286,667	270,854
PV	116,039	88,163	108,137	130,119	134,470	150,816	102,446
PV/AP (%)	39.6	51.4	50.8	51.8	51.5	52.6	37.8
PV/EC (%)	28.5	21.4	26.0	31.0	31.8	35.4	23.9
Congenital heart or lung diseases							
EC	—	—	—	—	—	—	927,946
Prevalence (%)	—	—	—	—	—	—	0.90
AP	—	—	—	—	—	—	189,317
PV	—	—	—	—	—	—	92,918
PV/AP (%)	—	—	—	—	—	—	49.1
PV/EC (%)	—	—	—	—	—	—	10.0
Chronic kidney disease							
EC	—	—	—	—	—	—	3,710,623
Prevalence (%)	—	—	—	—	—	—	3.60
AP	—	—	—	—	—	—	399,518
PV	—	—	—	—	—	—	228,147
PV/AP (%)	—	—	—	—	—	—	57.1
PV/EC (%)	—	—	—	—	—	—	6.1
Acquired immunosuppression							
EC	—	—	—	—	—	—	4,554,347
Prevalence (%)	—	—	—	—	—	—	4.40
AP	—	—	—	—	—	—	412,126
PV	—	—	—	—	—	—	157,059
PV/AP (%)	—	—	—	—	—	—	38.1
PV/EC (%)	—	—	—	—	—	—	3.4
Pregnant women							
AP	1,695,478	1,618,336	1,595,737	1,486,479	1,449,566	1,218,991	1,145,052
PV	1,002,151	1,035,901	1,106,145	1,119,098	1,063,026	1,014,659	949,523
PV/AP (%)	59.1	64.0	69.3	75.3	73.3	83.2	82.9
Health personnel							
PR	872,182	908,367	917,827	929,562	937,270	963,641	969,999
PV	838,262	898,488	1,007,047	1,086,328	776,616	1,042,618	888,637
PV/PR (%)	96.1	98.9	109.7	116.9	82.9	108.2	91.6

Morbid obesity was defined as body mass index (BMI) ≥ 40 kg/m^2^, according to the criteria established in the Mexican Universal Vaccination Program. Coverage indicators were calculated as the proportion of vaccinated persons relative to the corresponding estimated, notified, attended, or at-risk population. EC, estimated cases; NC, notified cases; AP, attended patients; PV, persons vaccinated; PR, personnel at risk.

Diabetes mellitus represented the most prevalent condition among individuals aged 5–59 years. Based on notified cases, the mean prevalence during the study period was 4.9% [Range: 4.7% (2015)–5.1% (2019)], corresponding to an annual average of 2,254,685 attended patients. However, prevalence estimates derived from ENSANUT data suggested a substantially greater disease burden, with an estimated mean prevalence of 7.2% [Range: 6.7% (2015)–7.6% (2018)]. Similarly, uncontrolled diabetes mellitus showed a mean notified prevalence of 3.4% [Range: 3.2% (2015)–3.5% (2019)], whereas epidemiological estimates based on ENSANUT data suggested an estimated prevalence of 4.9% [Range: 4.6% (2015)–5.2% (2018)].

Among individuals with diabetes mellitus, the mean influenza vaccination coverage was 45.1% [Range: 35.3% (2021)–58.9% (2020)] when considering notified cases, increasing to 60.4% [Range: 39.8% (2021)–68.1% (2020)] among attended patients. Vaccination coverage was consistently higher among individuals with uncontrolled diabetes mellitus, reaching 66.1% [Range: 51.7% (2021)–86.4% (2020)] relative to notified cases and 88.6% [Range: 58.4% (2015)–99.9% (2020)] among attended patients. Notably, uncontrolled diabetes represented 68.2% of the total attended diabetes population during the study period.

Morbid obesity (BMI ≥40 kg/m^2^), which is the obesity category formally included within the Mexican Universal Vaccination Program as an at-risk condition for seasonal influenza vaccination, showed a mean prevalence of 3.2% [Range: 2.7% (2015)–3.6% (2021)]. Influenza vaccination coverage in this population reached a mean of 58.9% [Range: 45.4% (2017)–74.4% (2020)] relative to the estimated population, whereas coverage among attended patients was substantially higher, with a mean annual coverage of 79.7% [Range: 77.1% (2016)–93.0% (2021)] during the study period.

Acquired immunosuppression represented one of the at-risk conditions most strongly associated with severe influenza outcomes and mortality (24). In 2021, the estimated prevalence of acquired immunosuppression in Mexico was 4.4%. Influenza vaccination coverage in this population reached 3.4% relative to the estimated population and increased to 38.1% among attended patients. Similarly, people living with HIV/AIDS were identified as another population at risk associated with unfavorable influenza-related outcomes (14). The estimated prevalence of people living with HIV/AIDS was 0.17% [Range: 0.15% (2015)–0.19% (2021)], although the true prevalence may be underestimated because of potential underreporting estimated between 30% and 90% (25, 26). Mean vaccination coverage among people living with HIV/AIDS was 38.5% [Range: 31.0% (2019)–45.9% (2021)], increasing to 49.8% [Range: 36.3% (2019)–56.5% (2017)] among attended patients.

Chronic kidney disease, another condition strongly associated with severe influenza outcomes and mortality (14), showed an estimated prevalence of 3.6% in 2021. Because mandatory notification of congenital heart or lung diseases, chronic kidney disease, and acquired immunosuppression was not implemented before 2021, complete epidemiological and vaccination records for these conditions were only available for the final year of the study period. Among attended patients, influenza vaccination coverage reached 57.1%, whereas coverage relative to the estimated population was only 6.1%.

Pregnancy represented another vulnerable population group associated with severe influenza-related complications for both the mother and the newborn (15). During the study period, influenza vaccination coverage among pregnant women reached a mean of 72.5% [Range: 59.1% (2015)–83.2% (2020)].

### 3.2 Distribution of influenza vaccine doses among target and at-risk populations

During the study period, a substantial proportion of influenza vaccine doses administered in Mexico were allocated to both the target population (TP) and the population at risk (PAR) (see Table 5). Between 2015 and 2021, an annual average of 22,914,563 doses [range: 20,623,099 (2021)–29,168,471 (2016)] was administered to the TP, whereas the PAR received an annual average of 11,023,190 doses [range: 9,253,662 (2017)–14,593,996 (2020)].

**Table 5:** Distribution of influenza vaccine doses administered among target and at-risk populations in Mexico, 2015–2021.

Overall influenza vaccine doses and population groups			
Year	Total doses administered	TP	PAR
2015	33,877,848	24,408,368	9,469,480
2016	38,819,552	29,168,471	9,651,081
2017	32,030,752	22,777,090	9,253,662
2018	32,730,814	21,695,634	11,035,180
2019	31,421,482	20,797,705	10,623,777
2020	35,525,571	20,931,575	14,593,996
2021	33,158,254	20,623,099	12,535,155

Distribution of administered doses among PAR groups (%)*	2015	2016	2017	2018	2019	2020	2021
Pregnant women	10.6	10.7	12.0	10.1	10.0	7.0	7.6
Health personnel	8.9	9.3	10.9	9.8	7.3	7.1	7.1
HIV/AIDS	0.6	0.5	0.8	0.6	0.5	0.5	0.7
Uncontrolled diabetes mellitus	18.1	21.4	22.8	22.2	24.8	20.6	14.4
Morbid obesity (BMI ≥40 kg/m^2^)	17.2	16.0	14.4	15.4	16.3	18.3	21.5
Cardiovascular disease	5.7	4.5	5.1	5.3	5.5	6.5	7.3
Chronic lung disease	7.8	6.8	5.7	5.6	6.0	6.2	8.5
Cancer	1.2	0.9	1.2	1.2	1.3	1.0	0.8
Congenital heart or lung diseases	—	—	—	—	—	—	0.7
Chronic kidney disease	—	—	—	—	—	—	1.8
Acquired immunosuppression	—	—	—	—	—	—	1.3
Other groups	30.0	29.8	27.2	29.8	28.3	32.9	28.3

TP, target population; PAR, population at risk; BMI, body mass index.

* Percentages may not total exactly 100% due to rounding and potential overlap among risk groups.

The proportional distribution of vaccine doses among PAR categories demonstrated marked variability across population groups. Individuals with uncontrolled diabetes mellitus consistently represented the largest proportion of administered doses among the defined PAR categories, accounting for a mean of 20.6% of doses distributed among PAR categories [range: 14.4% (2021)–24.8% (2019)]. Morbid obesity (BMI ≥40 kg/m^2^) represented the second largest proportion of distributed doses, accounting for a mean of 17.0% [range: 14.4% (2017)–21.5% (2021)].

Pregnant women and healthcare workers accounted for mean proportions of 9.7% and 8.6% of administered doses within the PAR, respectively. In contrast, people living with HIV/AIDS, cancer, cardiovascular disease, and chronic lung disease represented smaller proportions of administered doses throughout the study period.

Notably, individuals classified within the category of “other groups” represented a considerable proportion of administered doses, accounting for a mean of 29.5% [range: 27.2% (2017)–32.9% (2020)] of all doses distributed among the PAR. This category included individuals with risk conditions not specifically disaggregated within the available institutional databases.

As previously described, congenital heart or lung diseases, chronic kidney disease, and acquired immunosuppression were not separately reported in institutional vaccination records before 2021. Consequently, complete epidemiological and vaccination data for these conditions were only available for the final year of the study period.

### 3.3 Attended patients among populations at risk

**Figure 2:**
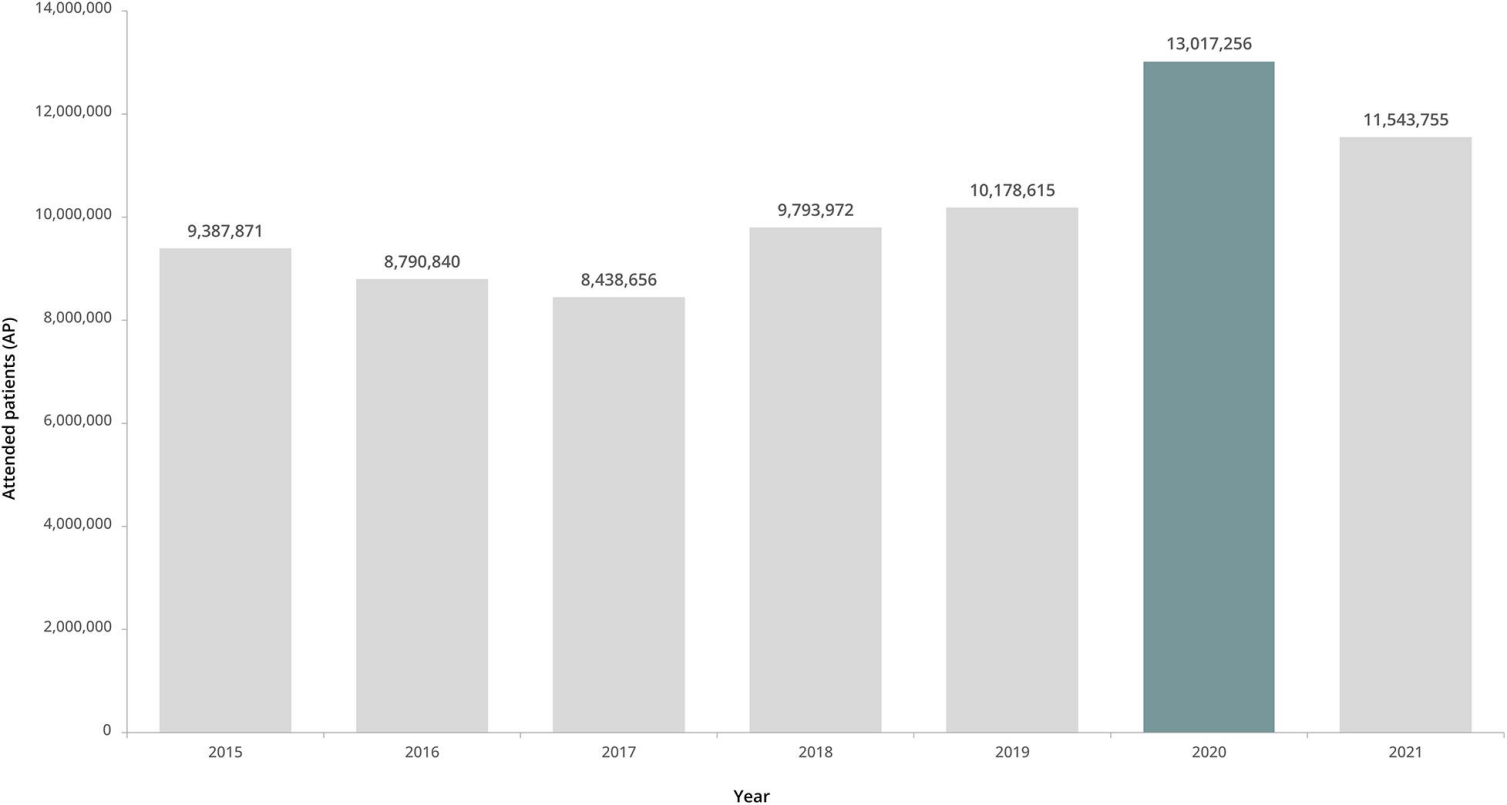
Temporal evolution of attended patients (AP) among populations at risk (PAR) in Mexico, 2015–2021. Annual number of attended patients registered among populations at risk eligible for influenza vaccination according to the Mexican Universal Vaccination Program. AP estimates correspond to individuals with at least one healthcare encounter during a given year, without duplication. AP, attended patients; PAR, population at risk.

Figure 2 shows temporal trends in attended patients (AP) among populations at risk (PAR) during the period 2015–2021. An annual average of 9,934,535 attended patients was observed during the study period [range: 8,438,656 (2017)–13,017,256 (2020)]. The highest healthcare demand was recorded in 2020, corresponding to the first year of the COVID-19 pandemic in Mexico.

The marked increase observed during 2020 likely reflects the substantial impact of the SARS-CoV-2 pandemic on healthcare service utilization, healthcare-seeking behavior, and institutional demand among populations at risk. This period was characterized by major operational changes within the Mexican healthcare system, including resource reallocation and increased pressure on healthcare services nationwide.

Individuals aged 20–49 years consistently represented the largest proportion of attended patients among populations at risk during the period 2015–2021, accounting for an annual mean of 43.5% [range: 41.2% (2015)–44.5% (2019–2021)]. This finding should be interpreted considering that the 20–49 years category encompasses a substantially broader population segment than the other age groups analyzed, spanning approximately three decades of life. This was followed by individuals aged 50–59 years, who represented a mean proportion of 38.5% [range: 38.0% (2016)–38.9% (2021)], despite encompassing only one decade of life.In contrast, younger age groups represented smaller proportions of attended patients throughout the study period. Individuals aged 5–9 years accounted for a mean of 8.1% of attended patients, whereas individuals aged 10–19 years represented a mean proportion of 9.9% of attended patients (Table 6).

**Table 6:** Distribution of attended patients (AP) among populations at risk (PAR) by age group and year in Mexico, 2015–2021.

Year	2015	2016	2017	2018	2019	2020	2021
Total attended patients (AP)	9,389,886	8,792,856	8,440,673	9,795,990	10,180,634	13,019,276	11,545,776
Distribution by age group (%)							
5–9 years	10.1	9.2	7.9	8.0	7.8	7.2	6.8
10–19 years	10.6	10.5	9.3	9.4	9.2	10.3	9.8
20–49 years	41.2	42.4	44.2	43.8	44.5	43.9	44.5
50–59 years	38.1	38.0	38.7	38.7	38.6	38.6	38.9

AP, attended patients; PAR, population at risk.

### 3.4 Forecasted healthcare demand among populations at risk

**Figure 3:**
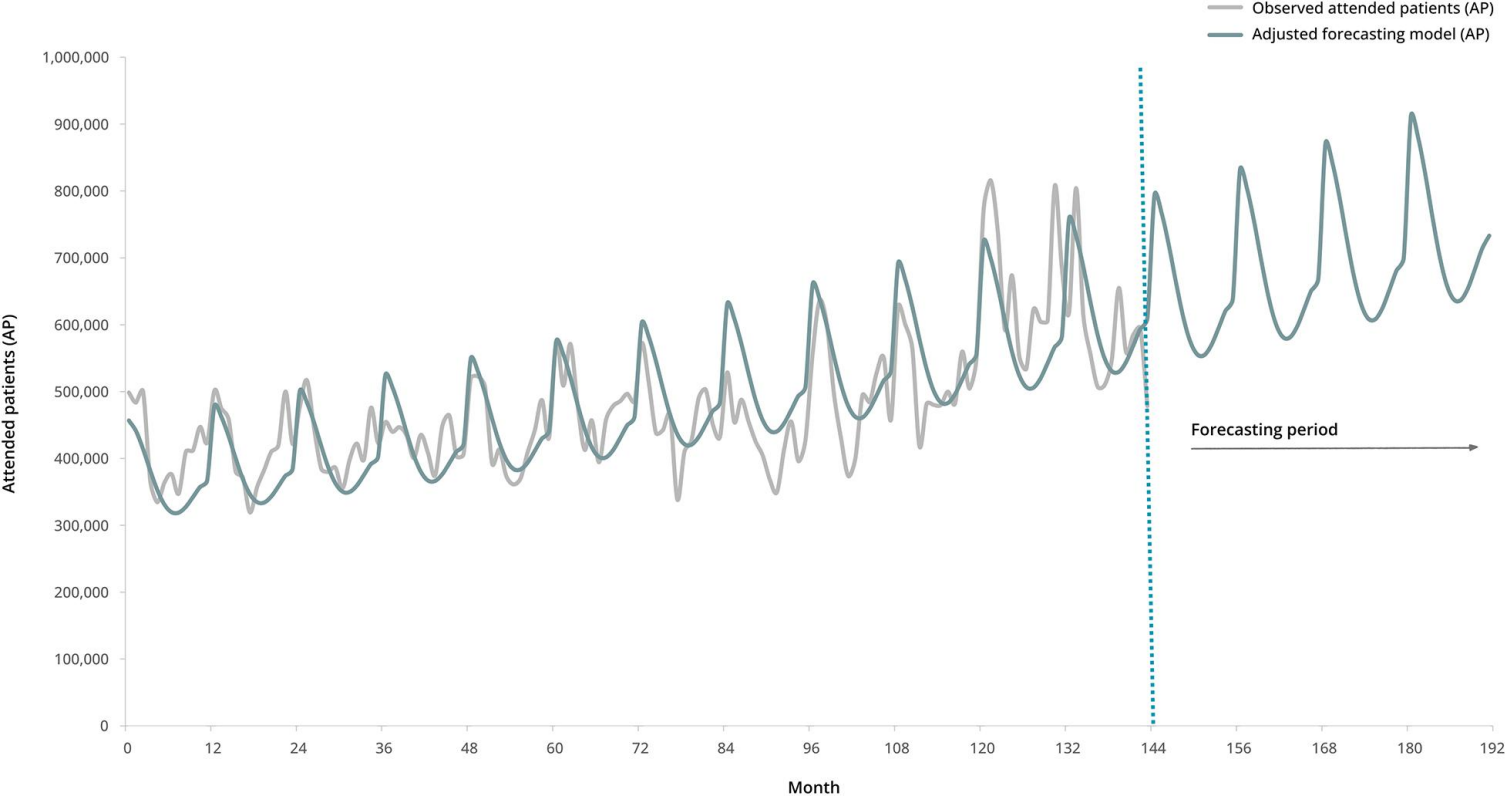
Adjusted forecasting model of attended patients (AP) among populations at risk (PAR) in Mexico during 2010–2021, with projected healthcare demand for 2022–2024. Observed attended patients are shown in green, whereas the adjusted forecasting model and projected estimates are shown in red. The vertical dashed line indicates the beginning of the forecast period. AP, attended patients; PAR, population at risk.

Forecasting analyses of attended patients (AP) among populations at risk (PAR) for the period 2022–2024 are presented in Figure 3. The adjusted time-series model projected a progressive increase in healthcare demand over the study period, with a more pronounced upward trend observed after 2018. This increase was primarily driven by high-prevalence comorbidities, particularly uncontrolled diabetes mellitus and morbid obesity.

The oscillatory pattern observed throughout the time series likely reflects variations in healthcare utilization, diagnostic activity, and institutional reporting dynamics rather than true epidemiological stability of the analyzed conditions. According to the adjusted forecasting model, the projected number of attended patients was estimated at 7,668,621 [95% CI: 7,531,581–7,805,661] for 2022, 8,030,366 [95% CI: 7,886,861–8,173,870] for 2023, and 8,805,853 [95% CI: 8,648,491–8,963,216] for 2024.

## 4 Discussion

In this study, we estimated the size of populations considered eligible for influenza vaccination due to at-risk conditions defined by the Mexican Universal Vaccination Program (PVU). To achieve this, administrative and epidemiological data sources from public healthcare institutions were integrated through the Mexican transparency and access-to-information framework. The resulting databases were standardized, organized, and analyzed using a duplicate-adjustment approach designed to minimize potential overlap among comorbidity categories.

A hierarchical population estimation framework was developed to characterize the potentially eligible population across different operational levels. Estimated cases (EC) represented the theoretical upper boundary of the eligible population based on epidemiological prevalence estimates derived from national and international sources. Notified cases (NC) corresponded to individuals with a documented diagnosis registered within healthcare system databases, whereas attended patients (AP) represented individuals with at least one healthcare encounter during a given year within the public healthcare system. Consistently across all analyzed populations at risk, the number of attended patients remained lower than the number of notified cases and substantially lower than the estimated population derived from prevalence-based projections.

Morbid obesity represents an illustrative example of the consistency of the proposed estimation framework. Because morbid obesity is a clinically identifiable and measurable condition, prevalence estimates derived from ENSANUT provided a reliable epidemiological reference. In this subgroup, the prevalence-based estimates and the attended population identified through healthcare records showed reasonable agreement. Although differences remained between both measures, this finding provides supportive evidence that the proposed framework generated epidemiologically plausible population estimates. Nevertheless, the results should be interpreted considering the limitations of administrative databases and the potential overlap among comorbidity categories.

Our findings also revealed a substantial vaccination gap among populations at risk. According to CONAPO demographic projections, approximately 103.2 million individuals aged 5–59 years lived in Mexico during 2021. In the same year, approximately 12.5 million influenza vaccine doses were administered among populations classified within the PAR. However, nearly 3.5 million doses corresponded to “other groups” not specifically classified within the analyzed at-risk categories. Consequently, only approximately 9 million doses could be specifically attributed to populations of primary interest, including approximately 0.89 million doses administered to healthcare workers.

Simultaneously, approximately 11.5 million attended patients with at-risk conditions associated with severe influenza outcomes and an additional 1.1 million pregnant women were identified within public healthcare institutions during 2021. These findings suggest that at least 4.6 million individuals at risk likely remained unvaccinated against influenza despite having active contact with the healthcare system during the analyzed year. Importantly, this represents a highly conservative scenario because the analysis only included individuals who actively utilized public healthcare services during that period.

Under this conservative framework, the estimated proportion of non-vaccinated individuals ranged from 7.0% among individuals with morbid obesity to 62.2% among individuals with cancer. Overall, the observed epidemiological and vaccination patterns remained relatively stable throughout the 2015–2021 study period.

The variability observed across PV/AP, PV/NC, and PV/EC ratios throughout the 2015–2021 period likely reflects the combined influence of epidemiological, operational, and healthcare-access factors affecting influenza vaccination coverage among populations at risk. Differences across analyzed conditions may be associated with variability in healthcare utilization patterns, diagnostic opportunities, disease awareness, frequency of healthcare encounters, and prioritization of vaccination strategies within public healthcare institutions. Temporal fluctuations may also reflect changes in vaccine availability, institutional vaccination capacity, public health campaigns, and healthcare system disruptions observed during the SARS-CoV-2 pandemic. Similar patterns were reported in Latin America, where influenza vaccination coverage increased substantially during 2020 in several risk groups, with healthcare worker coverage reaching approximately 100% in Argentina, Chile, and Uruguay before declining again during subsequent years (16).

Although the present study did not directly evaluate influenza-related clinical outcomes, these indicators may provide a useful operational approximation of potential vulnerability gaps among populations at risk. Lower PV/AP, PV/NC, and PV/EC ratios may indirectly identify subgroups with lower vaccination coverage relative to their estimated epidemiological burden and observed healthcare utilization, potentially reflecting populations at increased risk of preventable influenza-related complications, hospitalization, and mortality. Similar disparities in influenza vaccination coverage across high-risk populations have been reported previously, including persistent differences according to age, demographic characteristics, and the presence of high-risk conditions, even among populations with regular healthcare system access. In a large U.S. population-based study, overall influenza vaccination coverage ranged from 40.3% to 46.2% between 2017 and 2023 despite higher coverage among individuals with high-risk conditions (17). Consequently, these indicators may support future public health planning and prioritization strategies aimed at improving vaccination coverage among highly vulnerable populations.

Beyond the populations currently included within the Mexican Universal Vaccination Program (PVU), additional chronic conditions associated with severe influenza outcomes have also been recognized in international influenza vaccination recommendations, including those from the World Health Organization (WHO) (18). These include individuals living with disabilities, neurological or neuromuscular disorders, liver cirrhosis, hematological disorders, and certain congenital diseases.

The protective effect of influenza vaccination against hospitalization, severe disease, and mortality has been consistently documented in previous studies and international public health recommendations (18, 19). Moreover, the continuous antigenic evolution of influenza viruses contributes to substantial year-to-year variability in disease burden, including fluctuations in the number of cases, hospitalizations, and deaths (20). During the 2022–2023 influenza season in Mexico, one of the lowest lethality rates reported during the previous decade (∼3%) was documented; however, this season also recorded the highest number of laboratory-confirmed influenza cases (11, 20). Under these circumstances, maximizing vaccination coverage among populations at risk remains a key strategy to reduce healthcare system pressure and prevent avoidable morbidity and mortality. This becomes even more relevant considering the substantial socioeconomic burden associated with influenza-related disease (18, 19).

Low vaccination coverage among populations at risk is likely multifactorial. Contributing factors may include vaccine hesitancy, missed vaccination opportunities during healthcare encounters, limited awareness regarding vaccination recommendations, and programmatic or operational limitations affecting vaccine accessibility, availability, and timely distribution within public healthcare systems (21, 22). Previous studies have shown that healthcare access barriers, insufficient knowledge regarding vaccination, and operational challenges within immunization programs may substantially influence vaccination uptake among vulnerable populations (21, 22). Although Mexican public healthcare institutions have implemented strategies aimed at expanding influenza vaccination among populations at risk, our findings suggest that a considerable proportion of eligible individuals remain unvaccinated. Operational challenges associated with identifying specific vaccination-eligible conditions, such as morbid obesity, may also contribute to underrecognition of eligible individuals within routine vaccination practices.

An additional finding of interest was the large proportion of vaccine doses classified within undefined “other groups.” Further research is needed to better characterize these populations and to determine whether this pattern reflects healthcare-seeking behavior, institutional vaccination practices, administrative classification limitations, or changes in influenza epidemiology affecting younger adult populations during specific influenza seasons (11, 22). Previous studies have shown that influenza vaccination uptake may be substantially influenced by healthcare-access barriers, institutional practices, vaccine perceptions, and operational limitations affecting vaccination programs. A recent international review reported that lack of vaccine knowledge, negative attitudes toward healthcare, and limited trust in healthcare services were among the most frequently identified barriers associated with lower influenza vaccine uptake across adult populations (22).

Forecasting analyses presented in this study suggest a progressive increase in the size of populations at risk, consistent with the demographic and epidemiological transition currently occurring in Mexico, including population aging and the increasing burden of chronic non-communicable diseases undergoing (23). Consequently, the number of individuals potentially requiring influenza vaccination within the public healthcare system will likely continue increasing over time. These findings support the need for multilevel public health strategies aimed at improving vaccination coverage and optimizing vaccine allocation across different age groups, healthcare institutions, and at-risk conditions.

Beyond estimating vaccination gaps and the number of influenza vaccine doses potentially required among populations at risk, the analytical framework developed in this study may also support multiple public health applications. These data may contribute to healthcare resource planning, institutional preparedness strategies, and the identification of populations with limited vaccination coverage or restricted healthcare access. Additionally, the proposed operational indicators may support geographic and institutional prioritization strategies, monitoring of vaccination program performance over time, and future economic evaluations assessing the potential impact of expanding influenza vaccination coverage among vulnerable populations. The integration of epidemiological prevalence estimates with healthcare utilization patterns may also facilitate improved allocation of vaccines and healthcare resources within the Mexican public healthcare system, consistent with recent WHO recommendations aimed at strengthening national influenza vaccination programmes and improving vaccination policy implementation frameworks (24, 25).

This study has several limitations that should be considered when interpreting the results. First, the analysis relied primarily on administrative healthcare databases obtained from multiple public healthcare institutions, which may differ in coding practices, reporting completeness, and data quality. Second, ICD-10 administrative records do not provide sufficient clinical granularity to directly identify all vaccination-eligible subgroups defined by PVU criteria, therefore requiring the use of epidemiological prevalence estimates and operational assumptions to operationally estimate specific subgroups such as uncontrolled diabetes mellitus and morbid obesity. In addition, BMI measurements may not always be systematically assessed or recorded during routine vaccine administration, potentially leading to misclassification between obesity and morbid obesity within administrative registries. Third, despite the implementation of duplicate-adjustment methods, residual overlap among comorbidity categories may still persist due to the absence of fully interoperable patient-level databases across institutions. Additionally, some vaccination records were classified within undefined “other groups,” limiting the precise characterization of all vaccinated populations. Finally, forecasting analyses are inherently subject to uncertainty related to future epidemiological changes, healthcare utilization patterns, institutional reporting dynamics, and potential modifications in vaccination policies or influenza circulation patterns over time.

## 5 Conclusions

This study provides a comprehensive national-level estimation of populations eligible for influenza vaccination among at-risk populations in Mexico, integrating epidemiological prevalence estimates, administrative healthcare databases, vaccination registries, and healthcare utilization records. By combining prevalence-based estimates with healthcare system data, the proposed analytical framework allowed the identification of substantial gaps between the theoretically eligible population, the population actively utilizing healthcare services, and the population effectively vaccinated within the public healthcare system.

Our findings suggest that a considerable proportion of individuals at risk remain unvaccinated despite having active contact with healthcare institutions, highlighting important opportunities to strengthen influenza vaccination strategies among vulnerable populations. In addition, the forecasting analyses indicate that healthcare demand among populations at risk will likely continue increasing over time, consistent with the ongoing demographic and epidemiological transition observed in Mexico.

The study also highlights important operational and methodological challenges associated with estimating vaccination-eligible populations using administrative healthcare databases, particularly in relation to overlapping comorbidity categories, heterogeneous institutional reporting systems, and limited clinical granularity of ICD-10 registries. Nevertheless, the use of complementary epidemiological prevalence sources and duplicate-adjustment methods allowed the development of conservative and internally consistent population estimates.

During the preparation of this study, updated ENSANUT 2022 prevalence estimates for diabetes mellitus and obesity became available, reporting prevalences of 12.6% for diagnosed diabetes mellitus, 5.8% for undiagnosed diabetes mellitus identified during the survey, and 4.0% for grade III obesity among adults aged ≥20 years (26, 27). These updated estimates were broadly consistent with the epidemiological assumptions used in the present analysis and are unlikely to substantially modify the overall conclusions of the study.

Overall, the results of this study may contribute to future public health planning and support the optimization of influenza vaccination strategies targeting populations at risk within the Mexican public healthcare system.

## Data Availability

Publicly available datasets were analyzed in this study. The data used for this research were obtained through requests submitted to the transparency units of the relevant institutions; therefore, no direct links to the datasets are available.
