# Supplementary material for: Estimation of populations at risk for severe influenza and vaccination coverage in Mexico, 2010–2021
Source: Front Epidemiol. 2026 Jul 21;6:1826780. doi: 10.3389/fepid.2026.1826780 (PMC13433846; doi:10.3389/fepid.2026.1826780)
Supplement: Supplementary file 1 [file Table1.docx]

**Supplemental material**

Table A describes the variables included in each request, Table B lists the obligated subjects to which the information was requested by administrative level, name of the institution and response to the request, and Table C shows the data extracted for the prevalence estimates by population at risk.

**Table A.** Sources and values used for estimating prevalence in total population aged 5 to 59 years by population at risk.

| **Population at Risk** | **Source of the Data** | **Values** |
| --- | --- | --- |
| People living with HIV | HIV Epidemiological Surveillance System 2010 – 2021 | - Cumulative new cases detected: - 2015: 243,145 cases - 2016: 257,149 cases - 2017: 272,156 cases - 2018: 289,641 cases - 2019: 307,010 cases - 2020: 317,163 cases - 2021: 331,437 cases - (%) Cases living with HIV: 62.52% - (%) Cases living with HIV from 5 to 59 years: 95.6% |
| Diabetes Mellitus | Prevalence DM:  ENSANUT 2012, 2016, 2018 y 2021  Prevalence of uncontrolled DM: (Abreu, 2020) | - DM with Dx Previous (≥ 20 years):   - ENSANUT 2012: 9.2%   - ENSANUT 2016: 9.5%   - ENSANUT 2018: 10.2%   - ENSANUT 2021: 10.3% - DM sin Dx Previous (≥ 20 years):   - ENSANUT 2012: 3.8%   - ENSANUT 2016: 4.1%   - ENSANUT 2018: 4.9% - DM Total (≥20 years):   - ENSANUT 2016: 13.7% - DM con Dx Previous (10 to 19 years):   - ENSANUT 2012: Boys (0.60%); Girls (0.80%) - DM Uncontrolled: 68.3% |
| Morbid Obesity | Obesity Prevalence: ENSANUT 2012, 2016, 2018 y 2021  Morbid obesity prevalence:  (Barquera, 2020) | - 5 to 11 years:   - ENSANUT 2012: 17.4% (Men); 11.8% (Women)   - ENSANUT 2016: 18.3% (Men); 12.2% (Women)   - ENSANUT 2018: 20.1% (Men); 15.0% (Women)   - ENSANUT 2021: 23.8% (Men); 13.1% (Women) - 12 to 19 years:   - ENSANUT 2012: 14.5% (Men); 12.1% (Women)   - ENSANUT 2016: 15.0% (Men); 12.8% (Women)   - ENSANUT 2018: 15.1% (Men); 14.1% (Women)   - ENSANUT 2021: 21.5% (Men); 15.0% (Women) - ≥ 20 years:   - ENSANUT 2012: 26.8% (Men); 37.5% (Women)   - ENSANUT 2016: 27.7% (Men); 38.6% (Women)   - ENSANUT 2018: 30.5% (Men); 40.2% (Women)   - ENSANUT 2021: 31.8% (Men); 41.1% (Women) - Obesidad Mórbida (grado III):   - ENSANUT 2012: 2.9%   - ENSANUT 2018-2019: 3.6% |
| Cardiovascular disease | World Heart Federation/World Heart Observatory/Global Impact CVD/Mexico 2010-2021. | - 5 to 14 years:   - 2010: 0.5% (Men); 0.5% (Women)   - 2015: 0.5% (Men); 0.5% (Women)   - 2019: 0.5% (Men); 0.5% (Women) - 15 to 49 years:   - 2010: 1.5% (Men); 2.4% (Women)   - 2015: 1.6% (Men); 2.4% (Women)   - 2019: 1.7% (Men); 2.5% (Women) - 50 to 54 years:   - 2010: 5.8% (Men); 7.1% (Women)   - 2015: 6.2% (Men); 7.0% (Women)   - 2019: 6.1% (Men); 7.0% (Women) - 55 to 59 years   - 2010: 9.0% (Men); 10.0% (Women)   - 2015: 9.4% (Men); 9.9% (Women)   - 2019: 9.4% (Men); 9.8% (Women)   - ENSANUT 2021: 21.5% (Men); 15.0% (Women) - ≥ 20 years:   - ENSANUT 2012: 26.8% (Men); 37.5% (Women)   - ENSANUT 2016: 27.7% (Men); 38.6% (Women)   - ENSANUT 2018: 30.5% (Men); 40.2% (Women)   - ENSANUT 2021: 31.8% (Men); 41.1% (Women) - Obesidad Mórbida (grado III):   - ENSANUT 2012: 2.9%   - ENSANUT 2018-2019: 3.6% |
| Chronic Lung Disease including asthma and COPD | Global Burden of Diseases Study 2017. | Prevalence for Mexico (all chronic lung diseases): 6.14% |
| Cancer | Mexico Factsheet 2020 – Globocan | 5-year prevalence (all neoplasms): 0.41% |
| Congenital, chronic heart or lung disease, and other conditions that are associated with long-term use of salicylates | Global Burden of Diseases Study 2017. | Prevalence: 2.8% (includes congenital heart and lung malformations) |
| Chronic kidney disease | Perfil Epidemiológico de la Enfermedad Renal Crónica en México 2017. | Prevalence 2010: 5,331.01 x 100 mil hab.  Prevalence 2011: 5,440.30 x 100 mil hab.  Prevalence 2012: 5,575.79 x 100 mil hab.  Prevalence 2013: 5,724.03 x 100 mil hab.  Prevalence 2014: 5,890.37 x 100 mil hab.  Prevalence 2015: 6,076.35 x 100 mil hab.  Prevalence 2016: 6,283.73 x 100 mil hab. |
| Immunosuppression acquired by disease or treatment | Global Burden of Diseases Study 2017. | Prevalence: 4.4% (includes dermatological, gastrointestinal and musculoskeletal diseases). |

**Table B**. Obligated subjects and the responses via the Transparency Unit.

| **Administrative Level** | **Obligated subject** | **Response/Period** |
| --- | --- | --- |
| Federal Level | Secretaría de Salud (SSA) | Vinculo para descargar BD |
|  | Instituto de Seguridad y Servicios Sociales de los Trabajadores del Estado (ISSSTE) | Vinculo para descargar BD + Archivo en Excel |
|  | Instituto Mexicano del Seguro Social (IMSS) | Entrega de información en CD |
|  | Petróleos Mexicanos (PEMEX) | Entrega de información en CD |
|  | Secretaría de la Defensa Nacional (SEDENA) | Entrega de información en CD |
|  | Secretaría de Marina (SEMAR) | Entrega de información en CD |
|  | Instituto de Salud para el Bienestar (INSABI) | Archivo en Excel vía PNT |
|  | Centro Nacional para la Salud de la Infancia y la Adolescencia (CENSIA) | Archivo en Excel vía PNT |
| Estatal and Municipal Level | Instituto de Servicios de Salud del Estado de Aguascalientes | Archivo en Excel vía PNT |
|  | Secretaría de Salud de Aguascalientes | Archivo en Excel vía PNT |
|  | Instituto de Servicios de Salud Pública de Baja California | Archivo en Excel vía PNT |
|  | Secretaría de Salud de Baja California | Entrega de información en CD |
|  | Instituto de Servicios de Salud de Baja California Sur | Archivo en Excel vía PNT |
|  | Secretaria de Salud de Baja California Sur | Archivo en Excel vía PNT |
|  | Secretaría de Salud de Campeche | Archivo en Excel vía PNT |
|  | Instituto de Servicios Descentralizados de Salud Pública del Estado de Campeche | Archivo en Excel vía PNT |
|  | Secretaría de Bienestar de Campeche | Archivo en Excel vía PNT |
|  | Secretaría de Salud de Coahuila | Archivo en Excel vía PNT |
|  | Servicio Médico de los Trabajadores de la Educación de Coahuila | Archivo en Excel vía PNT |
|  | Servicios de Salud de Coahuila | Archivo en Excel vía PNT |
|  | Secretaría de Salud y Bienestar Social de Colima | Archivo en Excel vía PNT |
|  | Servicios de Salud del Estado de Colima | Archivo en Excel vía PNT |
|  | Secretaría-Instituto de Salud de Chiapas | Archivo en Excel vía PNT |
|  | Instituto Chihuahuense de Salud | Archivo en Excel vía PNT |
|  | Secretaría de Salud de Chihuahua | Archivo en Excel vía PNT |
|  | Secretaría de Salud de la Ciudad de México | Archivo en Excel vía PNT |
|  | Servicios de Salud Pública de la Ciudad de México | Archivo en Excel vía PNT |
|  | Secretaría de Salud del Estado de Durango | Entrega de información en CD |
|  | Secretaría de Salud de Guerrero | Archivo en Excel vía PNT |
|  | Secretaría de Salud del Estado de Hidalgo | Archivo en Excel vía PNT |
|  | O.P.D. Servicios de Salud Jalisco | Archivo en Excel vía PNT |
|  | Instituto de Salud del Estado de México | Archivo en Excel vía PNT |
|  | Instituto de Seguridad Social del Estado de México y Municipios | Archivo en Excel vía PNT |
|  | Secretaría de Salud del Estado de México | Archivo en Excel vía PNT |
|  | Secretaría de Salud de Michoacán | Archivo en Excel vía PNT |
|  | Secretaría de Salud de Morelos | Archivo en Excel vía PNT |
|  | Servicios de Salud de Morelos | Archivo en Excel vía PNT |
|  | Servicios de Salud de Nayarit | Archivo en Excel vía PNT |
|  | Servicios de Salud de Nuevo León, O.P.D. | Archivo en Excel vía PNT |
|  | Servicios de Salud de Oaxaca | Archivo en Excel vía PNT |
|  | Secretaría de Salud del Estado de Puebla-Servicios de Salud | Vinculo para descargar BD |
|  | Servicios de Salud del Estado de Querétaro | Archivo en Excel vía PNT |
|  | Servicios Estatales de Salud de Quintana Roo | Archivo en Excel vía PNT |
|  | Servicios de Salud de San Luis Potosí | Archivo en Excel vía PNT |
|  | Secretaría de Salud de Sinaloa | Sin respuesta |
|  | Servicios de Salud de Sinaloa | Archivo en Excel vía PNT |
|  | Servicios de Salud de Sonora | Vinculo para descargar BD |
|  | Secretaría de Salud Tabasco | Archivo en Excel vía PNT |
|  | Secretaría de Salud y Servicios de Salud de Tamaulipas | Archivo en Excel vía PNT |
|  | Secretaría de Salud y O. P. D. Salud de Tlaxcala | Archivo en Excel vía PNT |
|  | Servicios de Salud de Veracruz | Archivo en Excel vía PNT |
|  | Servicios de Salud de Yucatán | Archivo en Excel vía PNT |
|  | Servicios de Salud de Zacatecas | Vinculo para descargar BD |
| Healthcare Units | Instituto Nacional de Cancerología | Archivo en Excel vía PNT |
|  | Instituto Nacional de Cardiología Ignacio Chávez | Archivo en Excel vía PNT |
|  | Instituto Nacional de Ciencias Médicas y Nutrición Salvador Zubirán | Archivo en Excel vía PNT |
|  | Instituto Nacional de Pediatría | Archivo en Excel vía PNT |
|  | Instituto Nacional de Rehabilitación Luis Guillermo Ibarra | Archivo en Excel vía PNT |
|  | Hospital General "Dr. Manuel Gea González” | Vinculo para descargar BD |
|  | Hospital General de México "Dr. Eduardo Liceaga" | Archivo en Excel vía PNT |
|  | Hospital Infantil de México Federico Gómez | Vinculo para descargar BD |
|  | Hospital Juárez de México | Presencial UT |
|  | Hospital Regional de Alta Especialidad de Ciudad Victoria "Bicentenario 2010" | Archivo en Excel vía PNT |
|  | Hospital Regional de Alta Especialidad de Ixtapaluca | Archivo en Excel vía PNT |
|  | Hospital Regional de Alta Especialidad de la Península de Yucatán | Archivo en Excel vía PNT |
|  | Hospital Regional de Alta Especialidad de Oaxaca | Archivo en Excel vía PNT |
|  | Hospital Regional de Alta Especialidad del Bajío | Vinculo para descargar BD |
|  | Hospital Dr. Manuel Campos | Archivo en Excel vía PNT |
|  | Instituto Estatal de Cancerología de Colima | Archivo en Excel vía PNT |
|  | Instituto Estatal de Cancerología "Dr. Arturo Beltrán Ortega" de Guerrero | Vinculo para descargar BD |
|  | Hospital Civil de Guadalajara | Archivo en Excel vía PNT |
|  | Instituto Jalisciense de Cancerología | Archivo en Excel vía PNT |
|  | Hospital Regional de Alta Especialidad de Zumpango | Archivo en Excel vía PNT |
|  | Hospital del Niño Morelense | Sin respuesta |
|  | Hospital Central "Dr. Ignacio Morones Prieto" S.L.P. | Archivo en Excel vía PNT |
|  | Hospital Civil de Culiacán, Sinaloa | Archivo en Excel vía PNT |
|  | Hospital de la Madre y el Niño Guerrerense | Archivo en Excel vía PNT |
|  | Hospital de la Madre y el Niño Indígena Guerrerense | Archivo en Excel vía PNT |

**Table C**. Sources and data extracted for the prevalence estimates by population at risk.

| **Population at Risk** | **Source** | **Data extracted** |
| --- | --- | --- |
| People living with HIV | HIV Epidemiological Surveillance System 2010-2021 [1] | - New HIV cases detected and accumulated: 331,437 (until 2021). - Proportion of cases living with HIV: 62.52%. - Proportion of cases living with HIV from 5 - 59 yo 95.6%. |
| Diabetes Mellitus | - DM Prevalence [2], [3] - Uncontrolled DM Prevalence [4] | - DM with prior diagnosis (≥20 yo): 9.2% - 10.3%. - DM without prior diagnosis (≥ 20 yo: 3.8% - 4.9%. - Total DM prevalence: 13.7%. - DM with prior diagnosis (10 - 19 yo): Male (0.60%); Female (0.80%). - Uncontrolled DM: 68.3%. |
| Morbid obesity | - Obesity Prevalence [3] - Morbid Obesity Prevalence [5] | - Population from 5 - 11 yo: male (17.4% - 23.8%), female (11.8% - 15.0%). - Population from 12 - 19 yo: male (14.5% - 21.5%), female (12.1% - 15.0%). - Population from ≥20 yo: male (26.8% - 31.8%), female (37.1% - 41.1%). - Morbid obesity prevalence: 2.9% - 3.6% |
| Cardiovascular disease | - World Heart Federation/World Heart Observatory/Global Impact CVD/Mexico 2010-2021 [6] | - Population from 5 - 14 yo: male (0.5%), female (0.5%). - Population from 5 - 49 yo: male (1.5% - 1.7%), female (2.4% - 2.5%). - Population from 50 - 54 yo: male (5.8% - 6.2%), female (7.0% - 7.1%) - Population from 55 - 59 years: male (9.0% - 9.4%), female (9.8% - 10.0%). |
| Cancer | - Mexico Factsheet 2020 – Globocan [7] | - 5-years prevalence: 0.41% |
| Chronic kidney disease | Epidemiological Profile of Chronic Kidney Disease in Mexico 2017 [8] | - Prevalence: 5,331.01 - 6,293.73 cases per 100,000 inhabitants. |
| Congenital, chronic heart or lung disease, and other conditions associated with long-term salicylate use | - Global Burden of Diseases Study 2017 [2] | - Prevalence: 2.8% |
| Disease-acquired or treatment-acquired immunosuppression | Global Burden of Diseases Study 2017 [2] | - Prevalence: 4.4% (includes dermatological, gastrointestinal, and musculoskeletal diseases). |
| Chronic Lung Disease including asthma and COPD | Global Burden of Diseases Study 2017 [2] | - All chronic lung diseases: 6.14% |

*HIV: human immunodeficiency virus; DM: Diabetes Mellitus; CVD; Cardiovascular Disease, COPD: Chronic obstructive pulmonary disease; yo: years old.*

**Table D**. Description of the filtering method in coincident comorbidities (i.e., morbid obesity).

| **Demand of patients with obesity in the healthcare institutions (2021)** | **Cases** |
| --- | --- |
| Patients with obesity (A) | 27,119,580 |
| Patients with obesity (without coincident comorbidities) (B) | 26,091,576 |
| Patients with morbid obesity (without coincident comorbidities) (C) | 2,899,064 |
| Contraction factor to control duplications in morbid obesity (C/A) | 0.106 |

**Table E**. Variables included in requests for information to transparency units.

| **Variables** | **Description** |
| --- | --- |
| Population at risk using ICD-10 Codes | - People living with HIV (ICD-10: B20 - B24). - Diabetes mellitus (ICD-10: E10 - E14). - Obesity (ICD-10: E66). - Cardiovascular disease (ICD-10: I00 - 199, except essential arterial hypertension [I10X]). - Chronic pulmonary disease including asthma and COPD (ICD-10: J40 - J47). - Cancer (ICD-10: C00 - D48). - Congenital, chronic heart or lung disease, and other conditions associated with long-term salicylate use (ICD-10: Q20 - Q28 and Q30 - Q34). - Renal insufficiency (ICD-10: N17 - N19). - Disease-acquired or treatment-acquired immunosuppression (ICD-10: G60 - G64, G70 - G73, K50 - K52 and M00 - M99). - Pregnancy (ICD-10: Z34.9) |
| Groups of age | - 5 - 9 yo - 10 - 19 yo - 20 - 49 yo - 50 - 59 yo |
| Federative entity | All the states |
| Period of time | January 2010 – December 2021 |

*HIV: human immunodeficiency virus; ICD-10: International Classification of Diseases, Tenth Revision; yo: years old*

**References**

[1]	S. de S. S. de P. y P. de la S. D. G. de Epidemiología, “Informe Histórico Día Mundial VIH 2022,” 2022. [Online]. Available: https://www.gob.mx/salud/documentos/informe-historico-dia-mundial-vih-2022

[2]	S. L. James *et al.*, “Global, regional, and national incidence, prevalence, and years lived with disability for 354 Diseases and Injuries for 195 countries and territories, 1990-2017: A systematic analysis for the Global Burden of Disease Study 2017,” *Lancet*, 2018, doi: 10.1016/S0140-6736(18)32279-7.

[3]	M. Romero-Martínez *et al.*, “Encuesta Nacional de Salud y Nutrición 2018-19: metodología y perspectivas,” *Salud Publica Mex.*, 2019, doi: 10.21149/11095.

[4]	A. Basto-Abreu *et al.*, “Prevalencia de diabetes y descontrol glucemico en Mexico: Resultados de la Ensanut 2016,” *Salud Publica Mex.*, 2020, doi: 10.21149/10752.

[5]	S. Barquera and J. A. Rivera, “Obesity in Mexico: rapid epidemiological transition and food industry interference in health policies,” *Lancet Diabetes Endocrinol.*, 2020, doi: 10.1016/S2213-8587(20)30269-2.

[6]	World Health Organization (WHO), “Cardiovascular Disease Scorecards – Mexico,” 2022. [Online]. Available: https://world-heart-federation.org/wp-content/uploads/CVD-Scorecard-Mexico.pdf

[7]	The Global Cancer Observatory, “Globocan 2020: México,” *Int. Agency Res. Cancer*, 2020.

[8]	S. de S. S. de P. y P. de la S. D. G. de Epidemiología, “Perfil epidemiológico de la enfermedad renal crónica en México,” 2018.
